# Automated feedback and writing: a multi-level meta-analysis of effects on students' performance

**DOI:** 10.3389/frai.2023.1162454

**Published:** 2023-07-03

**Authors:** Johanna Fleckenstein, Lucas W. Liebenow, Jennifer Meyer

**Affiliations:** ^1^Digital Learning and Instruction, Department of Educational Science, University of Hildesheim, Hildesheim, Germany; ^2^Department of Educational Research and Educational Psychology, Leibniz Institute for Science and Mathematics Education, Kiel, Germany

**Keywords:** technology-based learning, automated writing evaluation, writing instruction, feedback, formative assessment, meta-analysis

## Abstract

**Introduction:**

Adaptive learning opportunities and individualized, timely feedback are considered to be effective support measures for students' writing in educational contexts. However, the extensive time and expertise required to analyze numerous drafts of student writing pose a barrier to teaching. Automated writing evaluation (AWE) tools can be used for individual feedback based on advances in Artificial Intelligence (AI) technology. A number of primary (quasi-)experimental studies have investigated the effect of AWE feedback on students' writing performance.

**Methods:**

This paper provides a meta-analysis of the effectiveness of AWE feedback tools. The literature search yielded 4,462 entries, of which 20 studies (*k* = 84; *N* = 2, 828) met the pre-specified inclusion criteria. A moderator analysis investigated the impact of the characteristics of the learner, the intervention, and the outcome measures.

**Results:**

Overall, results based on a three-level model with random effects show a medium effect (*g* = 0.55) of automated feedback on students' writing performance. However, the significant heterogeneity in the data indicates that the use of automated feedback tools cannot be understood as a single consistent form of intervention. Even though for some of the moderators we found substantial differences in effect sizes, none of the subgroup comparisons were statistically significant.

**Discussion:**

We discuss these findings in light of automated feedback use in educational practice and give recommendations for future research.

## 1. Introduction

Writing is a fundamental, versatile, and complex skill (Graham, 2019; Skar et al., 2022) that is required in a variety of contexts. Shortcomings in writing skills can thus hinder personal, academic, and professional success (Freedman et al., 2016; Graham et al., 2020). A basic aim of educational systems worldwide is to teach students to become competent writers; however, evidence suggests that while some students may achieve this goal, not all do (National Center for Educational Statistics, 2012, 2017; Graham and Rijlaarsdam, 2016). The situation is even further complicated by the fact that there is a large group of students from different language backgrounds who aspire to become competent writers in (English as) a second or foreign language and who are not always able to meet expectations (Fleckenstein et al., 2020a,b; Keller et al., 2020).

Writing skills are influenced by a variety of factors (Graham, 2018). Interindividual differences between writers are especially problematic as students with weak writing skills learn less in all school subjects compared to their more highly skilled classmates (Graham, 2019). In order to counteract this disadvantage, writing skills need to be promoted more in school. However, educational institutions often lack the time and personnel resources to do this. Graham (2019) reviewed 28 studies on writing instruction at school, identifying major indicators of inadequacy, including the insufficient instructional time devoted to writing (Brindle et al., 2015) and the absence of the use of digital tools for writing (Coker et al., 2016; Strobl et al., 2019; Williams and Beam, 2019).

In addition to high-quality, evidence-based teaching practice, digital technologies can be an asset in the individual promotion of writing skills. Automated writing evaluation (AWE) systems are able to assess students' writing performance, produce individualized feedback, and offer adaptive suggestions for writing improvement. Several individual empirical investigations have already looked into the employment of writing interventions with automated feedback tools, and some have investigated their effect on writing performance—with heterogeneous findings. Relevant moderators of effectiveness, however, have seldom been analyzed. The purpose of this study is to integrate the quantitative empirical literature on the subject of automated feedback interventions with a meta-analytic approach. Beyond the overall effect of automated feedback on student writing, we are particularly interested in moderating effects of learner and treatment characteristics.

## 2. Theoretical background

### 2.1. Formative assessment and AWE

Formative assessment serves to provide individualized learning support through a combination of (1) (standardized) learning progress evaluation, (2) individual task-related feedback, and (3) adaptive support for learners (Souvignier and Hasselhorn, 2018; Böhme and Munser-Kiefer, 2020). Implementing formative assessments is a challenge for educational systems, especially when it comes to higher-order competencies that require complex written responses from students. Assessing complex language performance as a necessary basis of individual feedback is a key challenge for teachers (Zhu and Urhahne, 2015; Fleckenstein et al., 2018). Especially judgment biases (e.g., tendencies toward leniency or severity; Jansen et al., 2019, 2021) and the use of simple heuristics in text assessment (e.g., text length; Fleckenstein et al., 2020a,b) can lead to inaccurate judgments of students' performance. Recent technological developments in the field of Artificial Intelligence (AI)—like AWE systems—can assist in the process of formative writing assessment.

The procedure of automatically scoring and evaluating students' written work through machine learning (ML) and natural language processing (NLP) techniques is known as automated writing evaluation (AWE; Bennett and Zhang, 2015). NLP is a subfield of AI that deals with the interaction between computers and humans using natural language. It involves the development of algorithms and systems that can understand, interpret, and generate human language. This includes ML algorithms, which learn from a large dataset of language examples and human ratings. When trained accordingly, AWE systems can evaluate a range of features of written text, including grammar, spelling, clarity, coherence, structure, and content. Based on these text features, they can assign scores to new texts and provide feedback to the writer (AWE feedback; Hegelheimer et al., 2016; Hockly, 2018).

AWE technology is utilized in a variety of educational contexts (Correnti et al., 2022), mainly for summative assessment purposes. Especially high-stakes standardized tests like the Graduate Record Exam (GRE) and the Test of English as a Foreign Language (TOEFL) have been using AWE technology for an automatic evaluation of students' writing (Zhang, 2021). In recent years, many tools have been developed to transfer this technology to low-stakes in-class writing tasks. The two major potentials of AWE with respect to formative assessment in writing are (a) assessment in terms of automatic evaluation of linguistically complex student responses and (b) individualized support through immediate and specific feedback based on students' performance. Various studies have demonstrated the quality of AWE assessment (Shermis, 2014; Perin and Lauterbach, 2018; Rupp et al., 2019; Zawacki-Richter et al., 2019). This review, however, focuses on the second part: Feedback that is based on the automated assessment. In the field of technology-supported writing instruction, this typically means supporting learners by providing adaptive automatic feedback on different textual aspects. While automatic assessment is not the central subject of this meta-analysis, it is the necessary foundation for adaptive feedback and individualized support. Therefore, automated assessment is an important inclusion criterion for the studies considered in this meta-analysis.

### 2.2. Feedback and AWE

Feedback is generally considered to be one of the most effective factors influencing student learning. This is not only shown by a solid empirical research base (*d* = 0.62; Hattie, 2022) but is also consistent with teachers' professional beliefs (Fleckenstein et al., 2015). For writing feedback in particular, a meta-analysis by Graham et al. (2015) showed effect sizes ranging from *d* = 0.38 to *d* = 0.87, depending on the source of the feedback. Despite these positive findings, process-oriented feedback, in particular, is rarely used by teachers in the classroom as it requires a lot of time and effort (Graham and Hebert, 2011). Feedback has a particularly positive effect on learner performance when it is given in a timely manner when it clarifies the gap between current performance and learning goal, when it reduces cognitive load, and when it is task-related, specific, and detailed (Mory, 2004; Hattie and Timperley, 2007; Shute, 2008; Black and Wiliam, 2009).

In the context of automated text evaluation, the quality of machine judgments is often evaluated on the basis of their agreement with human judgments. In terms of reliability and validity, many studies have come up with satisfactory results in this regard (Shermis, 2014; Rupp et al., 2019; Latifi and Gierl, 2021). Human raters do not necessarily outperform technology in all areas of text evaluation. With respect to segmenting and analyzing texts, experts tend to make coding errors, whereas with respect to recognizing relationships between concepts, human raters have been shown to be superior to technology (Burkhart et al., 2020). Moreover, both human and machine ratings can be affected by judgment bias in that certain text features are disproportionately included in the judgments (Perelman, 2014; Fleckenstein et al., 2020a,b).

Especially for writing complex and long texts, the evidence of the effectiveness of automated feedback has been described heterogeneously (Stevenson and Phakiti, 2014; McNamara et al., 2015; Strobl et al., 2019). In addition, Graham et al. (2015) noted that few randomized controlled experimental studies had been published. Review articles have either looked at the use of digital technologies in writing instruction in general (Williams and Beam, 2019; Al-Wasy, 2020) or focused on tools and how they work rather than their effectiveness (Allen et al., 2016; Strobl et al., 2019; Deeva et al., 2021).

More recent systematic reviews on the effectiveness of AWE feedback provided an overview of the relevant empirical studies and identified research gaps (Nunes et al., 2021; Fleckenstein et al., 2023). However, they did not quantify the effect of automated feedback on performance and, thus, could not empirically investigate the heterogeneity of effects.

Two very recent meta-analyses have examined the effect of AWE systems on writing performance (Zhai and Ma, 2021; Ngo et al., 2022). Ngo et al. (2022) performed a meta-analysis of AWE systems within the context of second or foreign language education. They found an overall between-group effect size of *g* = 0.59 and investigated several moderating variables, including publication data, population data, and treatment data. Zhai and Ma (2021) also included studies on first language writing in their meta-analysis and found an effect size of *g* = 0.86 for AWE on overall writing quality. However, as outcome measures, the authors included holistic scores only, leaving out individual components of writing performance. The authors found significant moderating effects of educational level, target language learners, and genre of writing.

## 3. Present study

Our meta-analysis goes beyond the scope of the previous meta-analyses concerning methodological and theoretical considerations. Like Ngo et al. (2022), we used a three-level model with random effects to perform the meta-analysis. However, whereas both previous meta-analyses included post-test data only, we included pre-test performance in the between-group analyses to achieve a more accurate effect size estimation (Morris, 2008). This is especially relevant when drawing on non-randomized primary data (i.e., quasi-experimental designs), for which an equal distribution of pre-test scores across groups cannot be assumed. Furthermore, we used robust variance estimation (RVE) to account for the dependence of effect sizes. Like Zhai and Ma (2021), we included L1 and L2 writers; however, we did not limit the range of outcomes and thus covered holistic and analytic measures of writing performance. We also investigated relevant moderators that have been neglected so far, including the type and level of outcome, the type of control condition, and the time of measurement.

This meta-analysis addresses the two following research questions:

RQ1: What is the overall effect of automated feedback tools on student learning based on an integration of primary studies?
RQ2: To what extent is the effect of automated feedback tools moderated by sample, intervention, and outcome characteristics?

## 4. Methods

### 4.1. Inclusion criteria

The analysis of the articles was conducted following the PRISMA (Preferred Reporting Items for Systematic Reviews and Meta-Analyses) model (Moher et al., 2009). This model provides an evidence-based minimum set of items for reporting reviews and meta-analyses. The selection and coding process for the articles was based on these standards.

In order to be included in the meta-analysis, studies needed to meet all of the pre-specified criteria regarding population, intervention, comparators, outcomes, and study design (PICOS) as specified below:

- Population: Students in primary, secondary and post-secondary education (ISCED level 1-7; UNESCO Institute for Statistics, 2012).
- Intervention: Automated writing evaluation (AWE) providing individualized or adaptive feedback to individual students.
- Comparators: Students receiving no feedback, non-automated feedback (e.g., teacher or peer feedback), or a less extensive form of AWE feedback.
- Outcomes: Writing performance (holistic or analystic) on a revision or transfer task.
- Study design: Experimental or quasi-experimental study designs with at least one treatment condition and one control condition.

Furthermore, studies had to be published in scholarly journals in order to be included. Studies investigating computer-mediated feedback by teachers or peers and studies on constructed responses in the context of short-answer formats were not considered in this meta-analysis.

### 4.2. Literature search strategy

The literature search was conducted in several literature databases (i.e., Ovid, PsycArticles, PsycInfo, Web of Science Core Collection, and ERIC), using various combinations of keywords: “automated writing evaluation;” “automated essay scoring;” writing + computer-assisted; writing + computer-based; writing + “intelligent tutoring system;” writing + “automated feedback;” writing + “electronic feedback;” writing + digital + feedback; writing + digital + scaffolding.

**Figure 1:**
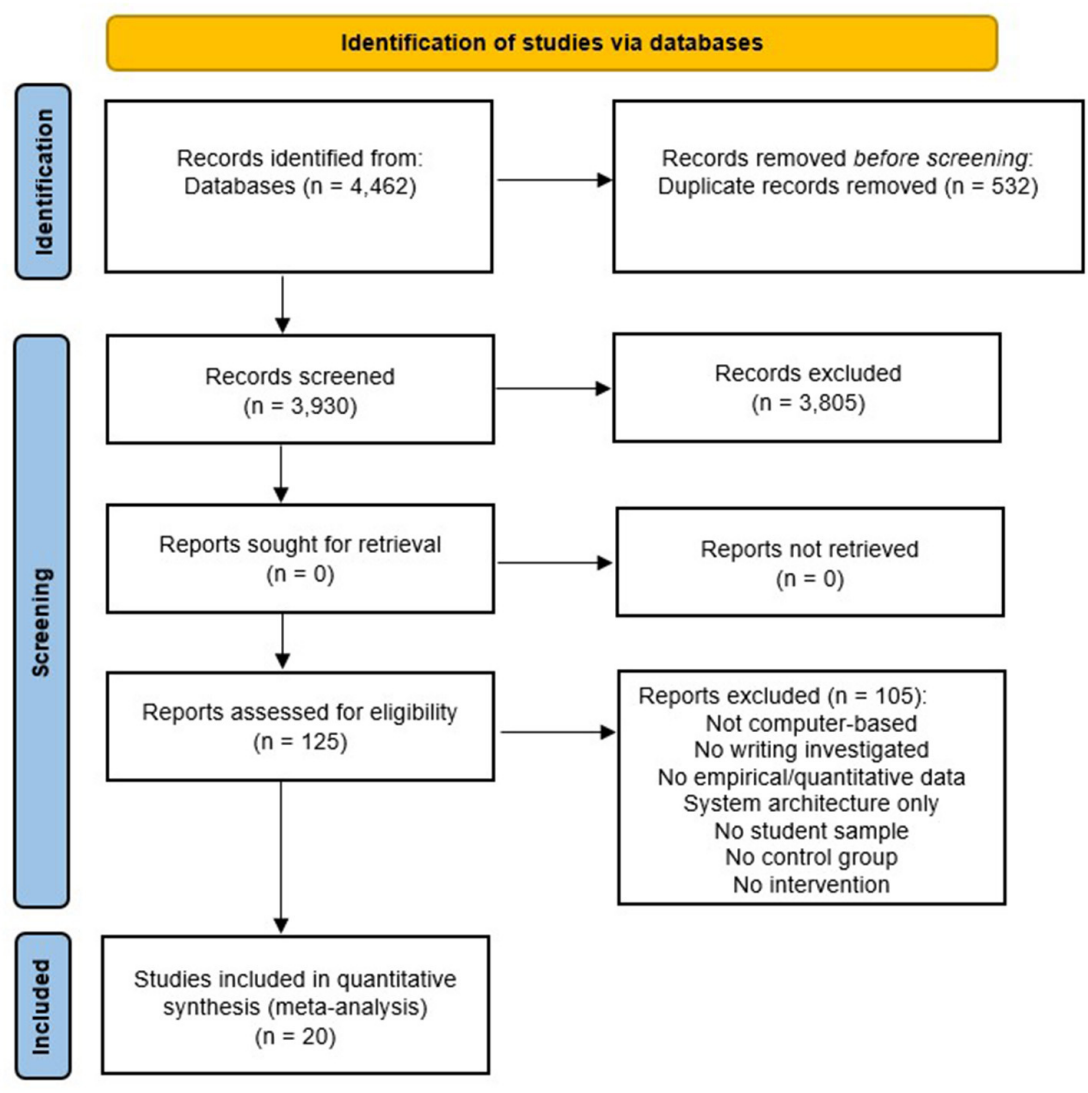
Flow-chart of the literature search and screening process (adapted from Moher et al., 2009).

The literature search yielded in a total of *N* = 4,462 reports. After removing duplicates, individual abstracts were screened using the open-source software ASReview (Van de Schoot et al., 2021) for screening prioritization. The tool uses Machine Learning to assist researchers in the process of reviewing large numbers of scientific abstracts. Active learning models iteratively improve their predictions in ordering the abstracts for presentation to the researcher. This procedure has been shown to reduce the number of abstracts to be screened to <40% while retaining a detection rate of 95% of the relevant publications (Ferdinands, 2021). So the goal of ASReview is to help researchers reduce the time and effort required to conduct a literature review, while also improving the quality and comprehensiveness of the review. Based on this, *n* = 125 full texts were screened, identifying *n* = 20 studies that met the inclusion criteria. Figure 1 provides an overview of the literature search and screening process according to the PRISMA guidelines. Following the identification of relevant studies, a coding scheme was developed, and all studies were coded by two independent coders. Any coding that differed was discussed and reviewed by the first co-authors of this paper and corrected if necessary. The variables that were coded and included in the moderator analyses are described in Section 4.5.

### 4.3. Effect size calculation

The standardized mean differences, also known as Cohen's *d*, between treatment and control conditions were calculated using the R package *esc* (Lüdecke, 2019). For studies that did not report raw statistics (e.g., means and standard deviation), we calculated Cohen's *d* based on other statistical indices (e.g., *F*- or *t*-values).

Morris (2008) recommended an effect size calculation based on the mean pre-post change in the treatment group minus the mean pre-post change in the control group, divided by the pooled pre-test standard deviation. This method was shown to be superior in terms of bias, precision, and robustness to the heterogeneity of variance. Thus, whenever pre-test values were available, they were considered in addition to the post-test values (also see Lipsey and Wilson, 2001; Wilson, 2016; Lüdecke, 2019). In further analyses, we conducted the same model but without considering the corresponding pre-test values to evaluate potential differences in results.

It has been found that Cohen's *d* tends to overestimate the true effect size when the study sample size is small (Grissom and Kim, 2005), which is the case in some of the included primary studies. Therefore, all Cohen's *d* values were converted into Hedges' *g*, which is an unbiased estimator that takes into account the sample sizes (Hedges, 1981):


$$\begin{array}{l} g=1-\frac{3}{4\left(n_{1}+n_{2}-2\right)-1}*d \end{array}$$

To verbally classify the effect sizes, we used a heuristic derived from the distribution of effects in this research field. This considered the 33rd and 67th percentile of the absolute value of all effects found in this meta-analysis: effects smaller than the 33rd were described as small, effects between the 33rd and 67th percentiles were described as medium, and effects greater than the 67th percentile were described as large (see Kraft, 2020, for a discussion on how to classify effect sizes; Jansen et al., 2022).

### 4.4. Meta-analytic integration of effect sizes

We combined the effect sizes of the included studies by applying a three-level model with random effects to take into account that several studies of our meta-analysis reported more than one effect size (Geeraert et al., 2004; Konstantopoulos, 2011; Cheung, 2014; Van den Noortgate et al., 2015; Assink and Wibbelink, 2016). This three-level model considers three levels of variance: variance of the extracted effect sizes at level 1 (sampling variance); variance between effect sizes of a single study at level 2 (within-study variance); and variance between studies at level 3 (between-study variance). Thus, this hierarchical model accounts for the variation of effect sizes between participants (level 1), outcomes (level 2), and studies (level 3).

The multilevel approach is a statistical approach that does not require the correlations between outcomes within primary studies to be known in order to estimate the covariance matrix of the effect sizes. Instead, the second level of the three-level meta-analytic model accounts for sampling covariation (Van den Noortgate et al., 2013). Also, the three-level approach allows for examining differences in outcomes within studies (i.e., within-study heterogeneity) as well as differences between studies (i.e., between-study heterogeneity). If a study reported multiple effect sizes from the same sample that could not be treated as independent from each other, we accounted for this non-independence by using the cluster-robust inference method (also called robust variance estimation; RVE; Sidik and Jonkman, 2006; Hedges et al., 2010; Tipton and Pustejovsky, 2015). This estimation allows for the integration of statistically dependent effect sizes within a meta-analysis without the need for knowledge of the covariance structure among the effect sizes. Furthermore, we conducted moderator analyses to test variables that may reduce within-study or between-study heterogeneity. For these analyses, the three-level random effects model can easily be extended by study and effect size characteristics into a three-level mixed-effects model.

The amount of heterogeneity (i.e., τ^2^), was estimated using the restricted maximum-likelihood estimator (Viechtbauer, 2005). In addition to the estimate of τ^2^, the *Q*-test for heterogeneity (Cochran, 1954) and the τ^2^ statistic (Higgins and Thompson, 2002) are reported. In case any amount of heterogeneity is detected (i.e., τ^2^>0, regardless of the results of the *Q*-test), a prediction interval for the true effect is provided (Riley et al., 2011). The regression test (Sterne and Egger, 2005), using the standard error of the observed outcomes as a predictor, is used to check for funnel plot asymmetry. The analysis was carried out using R (version 4.1.2; R Core Team, 2021) and the *metafor* package (Viechtbauer, 2010) to perform the meta-analyses. In addition, we used the *clubSandwich* package (Pustejovsky, 2022) to perform the cluster-robust inference method.

### 4.5. Moderation analyses

In combination with the consideration of heterogeneity in our data and calculated effect sizes, we performed several moderator analyses. Moderator variables can be used to provide a more meaningful interpretation of the data and reduce the heterogeneity of the overall effect. First, we identified possible moderator variables from the full texts of the primary studies: sample characteristics (educational level and language status); Intervention characteristics (treatment duration and type of control condition); outcomes characteristics (time of measurement, type of outcome, and outcome level). Second, the *n* = 20 studies included in the meta-analysis were coded by two authors of this study. Third, based on the final codes, the primary studies were divided into subgroups or factors that potentially explain the variance of the observed overall effect. In the following, the coded variables are explained in more detail.

#### 4.5.1. Sample characteristics

##### 4.5.1.1. Educational level

Studies that examined the effect of individual AWE feedback in high school (secondary level) were separated from studies that investigated higher education (tertiary level) students.

##### 4.5.1.2. Language status

As a sample characteristic, we coded language status into L1 for first or majority language contexts and L2 for second or foreign language contexts.

#### 4.5.2. Intervention characteristics

##### 4.5.2.1. Treatment duration

Interventions differed greatly in their duration, ranging from 50 min to one semester. Thus, we categorized intervention duration into short (one or two sessions) and long (more than two sessions).

##### 4.5.2.2. Type of control condition

The studies differed in their design with respect to the control group. In some studies, the control condition received no feedback of any kind on their writing; in other studies, the control condition received a different kind of feedback than the intervention group, such as teacher feedback, peer feedback, or a less extensive form of AWE feedback.

#### 4.5.3. Outcome characteristics

##### 4.5.3.1. Time of measurement

The reported effects were classified as either post-test performance (directly after the intervention) or follow-up performance (time gap between intervention and test.

##### 4.5.3.2. Type of outcome

Most studies on AWE feedback consider either the performance on a text revision or the performance on a different writing task. These outcomes differ in their conceptualization, as a successful revision can be considered performance improvement and a successful transfer to a new task can be considered learning.

##### 4.5.3.3. Outcome level

Furthermore, outcomes were categorized according to the level of detail. Outcomes were considered holistic when the effect referred to a total score or grade for the whole text. Analytic outcomes were further differentiated for effects concerning language aspects (e.g., grammar and mechanics) or content aspects (e.g., unity and number of subthemes) of the text.

## 5. Results

### 5.1. Overall effect of AWE feedback

A total of *k* = 84 effect sizes involving *N* = 2,828 learners from 20 studies were included in the analysis. The observed effects ranged from −1.73 to 2.99, with the majority of estimates being positive (70.24%). The estimated average effect size based on the three-level model with random effects was *g* = 0.55 (*SE* = 0.17) and differed significantly from zero (*z* = 3.18, *p* < 0.001). The comparison of the three-level model with the conventionally two-level model showed a significantly better fit for the three-level model, based on the likelihood ratio test (*X*^2^ = 150.36; *p* < 0.001). Therefore, the application of the three-level model would better explain the between-group comparison data.

According to the *Q*-test, the effect showed significant heterogeneity (see Table 1). The estimated variance values were τ^2^ for level 3 = 0.51 and τ^2^ for level 2 = 0.02. A 95% prediction interval for the estimated effect is given by −1.02 to 2.12. Hence, although the average effect is estimated to be positive, in some studies, the true effect may, in fact, be negative.

**Table 1:** Overall average effect size and heterogeneity test results including pre-test values.

**Weighted ES**			**95% CI**		**Heterogeneity**					
***k***	***g***	***SE***	**Lower**	**Upper**	***Q***	***df***	***p***	**I** ^**2**^_level3_	**I** ^**2**^_level2_	**I** ^**2**^_level1_
84	0.55	0.17	0.19	0.91	285.89	83	<0.001	81.37%	3.85%	14.78%

ES, effect size; CI, confident interval; k, number of effect sizes; g, Hedges' g standardized mean differences; SE, standard error.

As a further analysis, we examined whether the overall effect sizes differ when we ignore pre-test values from primary studies that provided them and calculated the effect sizes based only on the post-test values from the studies in concern (see Table 2). We observed an estimated average effect of *g* = 0.77 (*SE* = 0.20). The observed effects ranged from −1.14 to 3.61, with the majority of estimates being positive (70.24%). Therefore, the average effect differed significantly from zero (*z* = 3.87, *p* < 0.001). However, a Wald test showed that both effect sizes did not significantly differ from each other (*Q* = 0.67, *p* = 0.414).

**Table 2:** Overall average effect size and heterogeneity test results without pre-test values.

**Weighted ES**			**95% CI**		**Heterogeneity**					
***k***	***g***	***SE***	**Lower**	**Upper**	***Q***	***df***	***p***	**I** ^**2**^_level3_	**I** ^**2**^_level2_	**I** ^**2**^_level1_
84	0.77	0.20	0.35	1.18	985.01	83	<0.000	85,55%	9.71%	4.74%

ES, effect size; CI, confident interval; k, number of effect sizes; g, Hedges' g standardized mean differences; SE, standard error.

### 5.2. Publication bias

**Figure 2:**
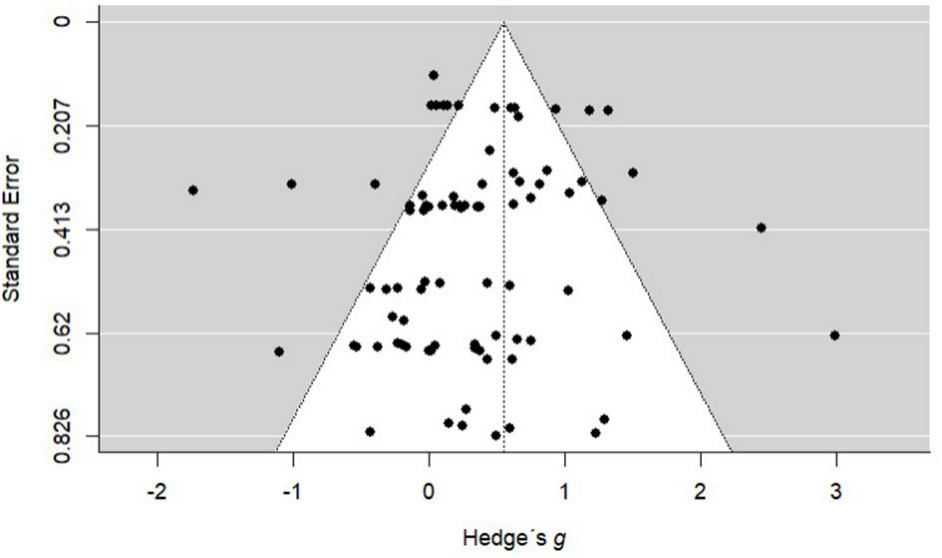
Funnel plot of all study effects.

To examine a publication bias, we used a funnel plot to see whether there is a symmetry of effect sizes, as they should be evenly distributed on both sides of the centered line, which represents the overall average effect sizes across all unique samples (Figure 2). In addition, we ran an Egger's test to evaluate the statistical significance of the asymmetry of the funnel plot by using the squared standard errors of the effect size estimates as a predictor in the meta-regression (Sterne and Egger, 2005). The results of the test confirmed that our funnel plot asymmetry is not different from zero (*b* = −0.76, SE = 0.69, *z* = −1.09, *p* = 0.27, 95% *CI* [−2.12 - 0.60]), indicating that there are no conspicuous data characteristics, producing an asymmetric inverted funnel plot. Therefore, we can assume the absence of a significant publication bias.

### 5.3. Moderation analysis

To test our hypotheses, we computed a random effects model with subgroup and regression effects of our coded moderator variables (see Table 3). For verbal classification, we used the 33rd percentile (*g* = 0.23) and the 67th percentile (*g* = 0.60) of the absolute values of the effects. Thus, effects below *g* = 0.23 were classified as “small,” effects between *g* = 0.23 and *g* = 0.60 were classified as “medium,” and effects above *g* = 0.60 were classified as “large.” The moderators were grouped into three categories: *sample characteristics, intervention characteristics*, and *outcome characteristics*.

**Table 3:** Moderation effects with *z*-tests against zero.

**Moderator**	**k**	**g**	**SE**	**z**	**df**	**p**	**95% CI**
**Sample characteristics**							
Educational level							
Secondary	23	0.50	0.16	3.13	4.95	0.03	[0.089; 0.921]
Tertiary	61	0.58	0.25	2.33	12.62	0.04	[0.039; 1.114]
Language status							
L1	58	0.40	0.12	3.28	8.86	0.01	[0.124; 0.676]
L2	26	0.72	0.35	2.08	8.71	0.07	[−0.066; 1.506]
**Intervention characteristics**							
Treatment duration							
Long	47	0.66	0.22	3.04	14.54	0.01	[0.196; 1.117]
Short	37	0.18	0.11	1.65	3.00	0.20	[−0.163; 0.514]
Type of control condition							
No feedback	68	0.59	0.15	3.84	14.70	0.00	[0.261; 0.912]
Other feedback	16	0.40	0.71	0.57	2.85	0.61	[−1.925; 2.73]
**Outcome characteristics**							
Time of measurement							
Post	66	0.57	0.17	3.44	18.43	0.00	[0.224; 0.926]
Follow-up	18	0.27	0.27	0.97	5.64	0.37	[−0.415; 0.947]
Type of outcome							
Performance	40	0.27	0.18	1.49	4.54	0.20	[−0.211; 0.755]
Learning	44	0.65	0.21	3.12	13.75	0.01	[0.203; 1.101]
Outcome level							
Holistic	29	0.50	0.22	2.30	16.91	0.04	[0.04; 0.965]
Content	25	0.57	0.18	3.20	13.84	0.01	[0.188; 0.952]
Language	30	0.61	0.17	3.61	12.00	0.00	[0.241; 0.975]

#### 5.3.1. Sample characteristics

##### 5.3.1.1. Educational level

We found medium effects for both secondary level (0.50) and tertiary level (0.58) that were both significantly different from zero. The difference between the two effects was not statistically significant (*Q* = 0.03, *p* = 0.854).

##### 5.3.1.2. Language status

For samples with the target language as L1, we found a medium effect (0.40); for those with an L2 background, the effect can be categorized as large (0.72). Both effects significantly differed from zero. The effects did not significantly differ from each other (*Q* = 0.82, *p* = 0.365).

#### 5.3.2. Intervention characteristics

##### 5.3.2.1. Treatment duration

Long interventions of more than two sessions showed a large significant effect (0.66), whereas short interventions of one or two sessions showed a small non-significant effect (0.18). However, the difference between the two effects was not statistically significant (*Q* = 1.32, *p* = 0.250).

##### 5.3.2.2. Type of control condition

For those Intervention groups that were compared to a control condition without any kind of feedback, the effect was significant and of medium size (0.59). When compared to a group with a different kind of feedback, the medium effect (0.40) was not significantly different from zero. However, the difference between effects was not statistically significant (*Q* = 0.16, *p* = 0.684).

#### 5.3.3. Outcome characteristics

##### 5.3.3.1. Time of measurement

For both post-test performance (0.57) and follow-up performance (0.27), we found effects that fall in the medium category. However, the follow-up effect did not significantly differ from zero, whereas the effect on post-test performance did. The difference between the effects was marginally significant on the 10%-level (*Q* = 2.71, *p* = 0.099).

##### 5.3.3.2. Type of outcome

The medium effect (0.27) for revision tasks as the outcome (performance) was not significantly different from zero. For transfer tasks (learning), the effect was large and significant (0.65). Again, the difference between effects was not statistically significant (*Q* = 1.75, *p* = 0.186).

##### 5.3.3.3. Outcome level

The effects of the three outcomes were all of medium-large size (holistic: 0.50; content: 0.57; language: 0.61), and they were all significantly different from zero. The three effects did not significantly differ from each other (*Q* = 0.51, *p* = 0.773).

## 6. Discussion

In the following, we discuss the central findings of this meta-analysis. Before we provide insight into automated feedback use in educational practice and give recommendations for future research, we briefly summarize our findings regarding the overall effect of AWE feedback and the moderator analyses.

### 6.1. Summary

This meta-analysis examined the overall effect of AWE feedback on writing performance by collecting 84 effect sizes from 20 primary studies with a total of 2,828 participants. A medium effect size of *g* = 0.55 was obtained using a three-level random-effects model. The findings support the use of AWE feedback to facilitate students' writing development.

The effect size is in line with prior meta-analytic research by Ngo et al. (2022), who found an overall between-group effect of *g* = 0.59. However, it is considerably smaller than the effect of *g* = 0.86 found by Zhai and Ma (2022). This variance in effect sizes may be due to the fact that the latter meta-analysis did not use a three-level model for their data analysis. Thus, they did not account for the dependence of effects reported within one study. They also did not include pre-test performance in their model; however, neither did Ngo et al. (2022).

Our robustness check showed that neglecting the pre-test performance in this research area could lead to an overestimation of the overall effect size (*g* = 0.77). However, this effect—although verbally classified as a large effect—did not significantly differ from the medium effect found in the original analysis.

Since the data showed significant heterogeneity, we investigated the impact of several potential moderators, including characteristics of the sample, the intervention, and the outcome. Even though for some of the moderators, we found substantial differences in effect sizes, none of the subgroup comparisons were statistically significant. This should be kept in mind when verbally classifying the effect sizes. In the following, we interpret our findings in light of previous research, especially the two recent meta-analyses by Ngo et al. (2022) and Zhai and Ma (2022).

Sample characteristics included the educational level and the language context. We differentiated for secondary and tertiary level, finding similar effects of medium size for both. This is contrary to the findings by Ngo et al. (2022) and Zhai and Ma (2022), who both found larger effects for post-secondary learners compared to learners at secondary level. However, both previous meta-analyses only included a very limited number of primary studies drawing on secondary-level samples (*k* = 3 resp. *k* = 6). Thus, it can be assumed that AWE feedback is similarly effective in both contexts. In terms of language context, the effect was large for L2 and medium for L1 contexts. Zhai and Ma (2022) reported a similar finding when comparing learners of English as a second or foreign language with native English speakers.

We found a large effect for long-term AWE feedback treatments but only a small effect for short interventions. This is in line with Ngo et al. (2022), who even found a small negative effect for short durations ( ≤ 2 weeks). The difference between medium and long intervention durations in Zhai and Ma (2022), however, was also not statistically significant. Zhai and Ma (2022) did also not find a significant effect for feedback combination (AWE only vs. AWE + teacher vs. AWE + peer). We took a slightly different approach and investigated different control conditions, some of which did not receive any feedback treatment and some of which received a different feedback treatment (e.g., teacher or peer feedback). Contrary to expectations, the medium-size effects did not differ significantly for the two types of control conditions.

Even though many studies in this field report post-test as well as follow-up outcomes, neither of the two prior meta-analyses investigated this as a moderator. We found the overall effect on post-test performance to be of medium size and not significantly different from zero; the effect on follow-up performance was small and did not significantly differ from zero. Again, in direct comparison, the difference between effects did not reach statistical significance. Neither of the previous meta-analyses looked into the type of outcome (i.e., performance vs. learning), even though this is a striking difference between studies that could explain the heterogeneity. To our surprise, the effect for revision tasks (performance improvement) was small, whereas the effect for transfer tasks (learning) was large. Only the latter differed significantly from zero. This indicates that AWE feedback does have an impact on learning to write rather than on situational performance enhancement. Unfortunately, the number of studies available does not suffice to investigate interactions of type of outcome with other moderator variables. Zhai and Ma (2022) only investigate holistic text quality as an outcome. Ngo et al. (2022) investigated outcome measure as a moderator with seven categories, finding effect sizes that ranged from *g* = 0.27 (Grammar and Mechanics) to *g* = 0.83 (Vocabulary). However, the effect sizes did not significantly differ from each other, probably due to small subgroup sizes. In our analysis of holistic and analytic (content, language) outcomes, we found very similar effects of medium size. More research on outcome measures as moderators of AWE feedback effectiveness is needed to investigate differential effects more closely.

#### 6.1.1. Limitations and directions for future research

Even though effects differed in size for some of the moderators, these differences were not statistically significant. Thus, the detected heterogeneity may be explained by variables other than the ones that we attended to in our moderator analyses. Thus, in future research, additional moderators need to be investigated. In other learning contexts, the type of feedback has been shown to moderate effectiveness (Van der Kleij et al., 2015; Wisniewski et al., 2020; Mertens et al., 2022). In the context of AWE feedback, we need more primary studies that compare different types of feedback or at least provide sufficient information on the details of their feedback intervention. Moreover, the design and presentation of automated feedback have rarely been investigated (for an exception, see Burkhart et al., 2020).

The potential to identify publication bias in a certain area of research is one of the strengths of meta-analytic research. We assessed publication bias by testing the asymmetry of the funnel plot, finding no indicator for bias. However, a more thorough analysis of publication bias is needed. In order to find out whether non-significant or small effects of AWE feedback tend to remain unpublished, the respective meta-analysis should include unpublished or non-peer-reviewed primary studies.

The variance in estimated effect sizes across AWE feedback meta-analyses calls for a second-order meta-analysis. The purpose of a second-order meta-analysis is to estimate the proportion of the variance in meta-analytic effect sizes across multiple first-order meta-analyses attributable to second-order sampling error and to use this information to improve the accuracy of estimation for each first-order meta-analytic estimate (Schmidt and Oh, 2013). Thus, a second-order meta-analysis would inform AWE feedback research and provide a more comprehensive understanding of factors influencing AWE feedback effectiveness.

### 6.2. Practical implications

This meta-analysis showed that AWE feedback has a medium positive effect on students' writing performance in educational contexts. However, the heterogeneity in the data suggests that automated feedback should not be seen as a one-size-fits-all solution, and its impact may vary based on factors such as context and learner characteristics, the feedback intervention itself, and outcome measures.

For teachers and school administrators, this implies that AWE feedback can be a useful tool to support students' writing in educational contexts, but its use should be carefully considered and integrated into a comprehensive approach to writing instruction. The use of automated feedback should be combined with other forms of support, such as teacher feedback and individualized learning opportunities, to ensure its effectiveness.

Furthermore, the heterogeneity in the results suggests that automated feedback may not have the same impact on all students. Teachers and administrators should consider the individual needs and characteristics of their students when deciding whether and how to implement automated feedback. Further research is needed to determine the most effective use of automated feedback in different educational contexts and with different populations. Teachers and administrators should keep up to date with developments in the field and use evidence-based practices to inform their decisions.

## Data availability statement

The original contributions presented in the study are included in the article/supplementary material, further inquiries can be directed to the corresponding author.

## Author contributions

JF, LL, and JM contributed to conception and design of the study. JF and LL conducted the literature search, screening procedure, and coding. LL performed the statistical analysis and wrote sections of the manuscript. JF wrote the first draft of the manuscript. All authors have read and approved the submitted version.
